# Lecture on Tooth-Ache

**Published:** 1845-06

**Authors:** Edwin Saunders

**Affiliations:** Surgeon Dentist to St. Thomas's Hospital, and to the London Institution for Diseases of the Teeth, &c. &c.


### ARTICLE III.

*Lecture on Tooth-Ache.* Delivered at St. Thomas's Hospital, in the Session of 1844-5, by EDWIN SAUNDERS, Esq., M. R. C. S., Surgeon Dentist to St. Thomas's Hospital, and to the London Institution for Diseases of the Teeth, &c. &c.

GENTLEMEN—

IN fulfilment of our design of being strictly practical in the present course, and of giving priority of consideration to those diseases of the teeth, and their treatment, which are of the most frequent occurrence; I shall now request your attention to that painful malady, to which few, indeed, are strangers, known as odontalgia or tooth-ache. As, upon this subject, considerable misapprehension prevails, arising from the confusion of several affections to which these organs and the structures with which they stand in more or less close relation are liable, it will be necessary to recur for a moment to the anatomy of the parts, in order to a more perfect discrimination of their pathological conditions. Under the name of tooth-ache, in its common acceptation, are comprehended all pains, whether of an acute or chronic character, whether diffused or concentrated, which arise from, or are supposed to be dependent upon, a diseased condition of the teeth, or their lining, or enveloping structures. When we come to examine this subject more closely, however, we shall see that as it may arise from totally distinct pathological conditions, so it admits and demands a diversity of treatment. If the same amount of patient and careful examination, and of philosophical acumen were brought to bear upon this very prevalent and distressing malady, as are enjoyed by other, and scarcely less valuable organs, we have no hesitation in asserting that it would be not only divested of much of that mystery in which its source and nature are now veiled, but would be found to be amenable, in a great degree, to ordinary and accessible remedial means. There is, probably, no one malady to which the human frame is liable, the treatment of which is more undefined and empirical, and yet there is none which more frequently, and with more heart-touching appeals, demands the best offices of the practitioner of the healing art. How often is the surgeon dismayed by seeing his patient already prostrated and enfeebled by disease, racked by this relentless and tormenting malady. The extreme depression of the whole nervous system, consequent upon sleepless nights and days of agony, makes his patient shrink with horror from the violent but only remedy on which he can with confidence rely; while the hourly increasing febrile excitement gives rise to the most alarming symptoms. The glazed and anguished eye reproachfully implores him to propose some less vehement means of alleviating his misery, and recourse is had to so called remedies, empirical in character, uncertain and contradictory in effect, and which only issue in a needless exacerbation of symptoms. Of what incalculable advantage, under such circumstances, to be able accurately to diagnose symptoms, and by the felicitous application of appropriate remedies, to mitigate present suffering, and provide against a recurrence of mischief! Even in health, and in more favorable cases, how immensely superior in the estimation of his patient, will be the practitioner who is able, from previous acquaintance with the subject, to combat a malady which prostrates the boldest, and incapacitates the most resolute for the ordinary business and duties of life! Justly, indeed, though with more of vigor than elegance of expression, has Burns, in his ode, characterised the tooth-ache, as "Thou hell o' a' diseases."

The treatment of this malady appears to have been but imperfectly understood by the ancients, their chief reliance, as in most cases of occult and acute disease, more especially when connected with the nervous system, being placed upon certain nostrums and charms. Aretæus, with commendable candor, says, the cause of tooth-ache is known to the gods alone. And, indeed, when we contemplate the variety of remedies which have been at various periods proposed as specifics, and the number which are still held in estimation by various persons, both in and out of the profession, as well as the avidity with which any new nostrum is received at the present time, it might seem that science has even now afforded but little additional light upon the subject. Scarcely a single therapeutic agent could be named, which has not, at some period, been enlisted, in some form or other, in this cause. The whole class of stimulants—the essential oils—the family of narcotics—mineral acids—vegetable alkalies—escharotics—cantharides—-galvanism—-mesmerism—smoke—vapor—the cautery—friction—homœopathy, and hydropathy, have all their votaries, even at the present time, and are all, in their turn, recommended and employed, with (it may be) an unreasoning, but withal, a devoted faith, to which many of them are, doubtless, mainly indebted for their efficacy. Be it our aim, now to seek to eliminate from this heterogeneous mass the grain of gold, or, to use another figure, to extricate from the shapeless rock, the sculptured beauty which therein lies entombed, sustained by the assurance, that if we faithfully and diligently apply our powers of observation, the means of combating disease, alike in its mildest and its direst forms, lie about us, and within our reach.

If we examine a tooth, anatomically, we shall find it to consist, or to be in relation with the following structures. First, the crown of the tooth, with its external envelope of enamel: the root, which is inserted into its appropriate cell, or alveolus, covered with its investing, or peridental membrane; and the neck, or that part which is situated at the junction of these, and is embraced by the gum. If, now, we make a longitudinal section of the tooth, a cavity (cavitas pulpæ) will be descried in the centre, partaking of the general form of the tooth, being largest in the crown and neck, gradually diminishing in size through the root, or roots, and terminating in a minute canal. This cavity, which is nearly equidistant every where from the surface, contains the highly vascular pulp, the remnant of the formative organ of the ivory or dentine, and which is destined for its nourishment and the maintenance of its vitality. This pulp, which is made up of a reticulated plexus of vessels and nerves, united together by a cellular basis, is possessed of exquisite sensibility, and is exceedingly prone to inflammation on the slighest pressure or exposure. The membraneous envelope of the root is also liable to inflammation, acute and chronic, symptomatic, or as a consequence of long continued irritation of the pulp; or, purely idiopathic, arising from cold or injury. Here, then, we have two obvious sources of odontalgia, or pain connected with teeth. We say nothing now of a morbid sensibility of the teeth, or of their membranes, produced by absorption of the gums and alveoli, confirmed dyspepsia, the exhibition of mercury, or of acid medicines, nor of the pseudo, or purely symptomatic tooth-ache, so frequently experienced during pregnancy. For the present we will consider the true tooth-ache arising from local causes and not dependent upon a constitutional origin.

From what we have seen, then, this painful affection may arise either from exposure of or pressure upon, the internal pulp, or from lesion of the investing or peri dental membrane, and these two sources of pain are accompanied and distinguished by different and appropriate symptoms. The most prevalent form of tooth-ache in early life is the acute, and arises from the former source. It is characterised by a sharp lancinating pain, of sudden accession, of limited duration, (for if continued it would soon exhaust the powers of life,) and is usually to be traced to a carious condition of the organ in which it occurs. It is ordinarily referable to some definite and assignable cause, such as sudden fracture of the carious and fragile shell of enamel, the impaction in the carious cavity of some particle of food, or exposure to cold and currents of cold air. Thus a tooth which has been felt to be unsound, but has never been the cause of more than temporary uneasiness, becomes, during the act of mastication, suddenly broken down, and portions of food and enamel are thrust upon the highly organised and sensitive pulp. The immediate effect of this is to set up inflammation more or less intense, varying with the extent of lesion of the organ or with the degree of nervous excitability of the individual. This inflammation, which is usually sufficient to bring on a paroxysm of that peculiar, concentrated and intense pain, known as tooth-ache, it is almost impossible to subdue or even materially alleviate. The highly vascular pulp which, in its healthy or normal state, occupies, completely, the whole of the circumscribed and unyielding cavity already described as existing in the centre of the tooth, being now engorged with an increased supply of blood, seeks to relieve itself by swelling as in other cases of simple and acute inflammation. But being pent up within a limited and inexpansible boundary becomes, in its turn, still further pressed upon in every direction, the symptoms being aggravated by the impaction of some foreign body in the only direction in which even partial relief was possible.

With this view of the causes and pathology of tooth-ache, we are no longer under a necessity of accounting for the intensity of the pain, by attributing to the tooth-pulp a specialty of function or of sensibility; the circumstances in which it is founded being fully adequate to explain the agony with which it is accompanied. In all probability, if we had the opportunity of applying in these the mode of treatment adopted in cases of ordinary inflammation, occurring in structures more favorably circumstanced, we should be able at least to mitigate its symptoms; if we were in possession of the means of depletion and the removal of pressure, so as to avoid tension, much not only of suffering but of eventual mischief might be obviated. But as this is impossible, some other and more energetic mode of treatment is called for. Caustic, the most active stimulants, oil of cloves, creosote, the actual cautery, and the more powerful escharotics as chloride of zinc, the common white oxyde of arsenic are, in such cases, valuable and favorite remedies. These, of course, act immediately by destroying the vitality, and, consequently, the sensibility of the exposed pulp. To enumerate, indeed, the variety of remedial agents employed for this purpose, would far exceed the limits of this lecture, and would rather distract the attention than lead to any useful practical result.

In the treatment of cases of acute tooth-ache, arising from exposure of the pulp, two methods present themselves, the one by the reiterated application of stimulants to exhaust the sensibility of the part, a slow and somewhat uncertain proceeding, frequently issuing in disappointment to the practitioner, and impatience on the part of the sufferer; and the other, by causing, through the employment of more active means, more quickly and scarcely with more pain the death of the part. With the former intention the camphorated spirit or Eau de Cologne are frequently and with various success employed; indeed, as benefit is derived from varying the stimulant, the alternate application of both on small pledgets of lint is to be recommended. In this mode of treatment it will be found of the utmost consequence not to overload the cavity, more especially if this should be interstitial, as not only would an injurious pressure be made on the morbidly sensitive structure beneath, which, in the latter case would be productive of periosteal inflammation in the affected, as well as in the adjoining tooth, but the stimulant, instead of reaching the destined spot, would be pressed out on its introduction to the cavity. The length of time necessary to effect the object in this case—the exhaustion of the sensibility, or the absorption of the pulp—will depend upon the extent of surface exposed. It may be effected within two or three weeks, or it may require more than as many months, the application being renewed two or three times in the twenty-four hours. There are cases in which this somewhat tedious process is preferable, on account of its gradual and almost painless nature; and in which a more active treatment, from the peculiarity of temperament or constitution, would involve an amount of pain which could ill be sustained. Ordinarily, where the cavity is kept continually guarded by some such application, no pain is experienced beyond a slight thrill on its first introduction, and it has the further advantage of condensing and rendering insensible the parieties of the cavity, so as to render it fit for the ultimate measure of stopping. For such the camphorated solution of mastic will be found a convenient and effectual preparation.

Under more favorable circumstances, however, and where the mode of treatment is left to the election of the operator, a more energetic course will be adopted. If the pain be violent, he will gently, and with extreme care, wipe out the cavity with soft cotton wool, or the more pulpy portion of lint, he will thus be enabled to detect the presence of any foreign matter, which he will cautiously remove; he will then insert some anodyne, camphor and opium, the muriate or acetate of morphia, or simply a little wool saturated with the tinct. opii. This will allay present pain, but he will be anxious to do more, and in order to do this, he will, with a sharp cutting instrument, remove a layer of carious bone, and at the same time by a sharp and dexterous movement, will excise the exposed surface of the pulp. This may be effected with little pain, if done unhesitatingly and with decision, nay, in most cases, without the knowledge of the patient. We have now a bleeding instead of an inflamed and highly tense surface, and if we now apply a few granules of the white oxyde of arsenic, an eschar is formed in the course of a few hours, which will be totally insensible and capable of sustaining pressure. The cavity in this case should be well guarded by being sealed up with wax, and it should be retained for not less than six hours. When thus treated, but little pain will be experienced, in the majority of cases, not amounting to more than uneasiness, and this of half an hour's duration. In less fortunate cases, however, the pain does not acquire the intensity of ordinary tooth-ache, and is found to be entirely subdued by copious warm fomentations. When this previous excision is omitted, and the arsenic is applied directly to the already inflamed surface, much more acute suffering is induced, and in such cases it is usual to combine with the escharotic some anodyne effect. For this purpose, morphine has been employed in various proportions; some practitioners, however, and I confess myself amongst the number, prefer to apply the arsenic on a pledget of wool or lint, which has been previously dipped in creosote.

This, then, constitutes the radical treatment of the acute form of tooth-ache, and is preliminary to the restoration of the organ to its wonted function, by the very beautiful and valuable operation now so well understood and so generally employed, termed stopping, and to which we shall hereafter refer. Other remedial agents, however, than these here pointed out, are occasionally employed, and as a fair and full examination of these cannot but be most valuable to the cause of dental surgery, they may well merit attention in this place. Amongst the most valuable is, perhaps, the chloride of zinc, although my own experience of its effects would lead me, in most cases, to prefer the former remedy, (for the introduction of which we are indebted to our transatlantic brethren, whose zeal in the prosecution of this department of the healing art deserves more than a passing tribute,) as being more uniform and certain in its action. Pure tannin or tannic acid has been employed with success by Mr. Tomes, but, judging from some cases, insufficient, perhaps, to justify any very decided opinion, it appears to be inadequate to produce the desired effect. The nitrate of silver is less energetic in its action, requiring repeated applications, and is open to the objection of blackening the tooth. Nitric acid has been employed, but has deservedly fallen into disuse, from its destroying the parieties of the cavity, by taking up the earthy matter of the dentine. The actual cautery is still a favorite instrument with French practitioners, but the difficulty, amounting almost to impossibility, of preventing the temperature from being lowered by the air and vapor of the mouth in its passage to the tooth, not to mention the difficulty of applying it to the denuded spot, have led to its general abandonment in this country. Creosote, as a remedy in ordinary tooth-ache is, undoubtedly, valuable, but, to be efficacious, it should be employed with care, and on the fresh exposure of the pulp. In cases of chronic inflammation, or where the pulp has been frequently exposed to irritation, it will, I believe, be found of little use. In all cases it is important to carefully dry and cleanse the cavity from any foreign matter, in order to prevent its dilution and to ensure its coming in contact with the affected surface. An old and favorite remedy, and one which, as far as temporary alleviation of symptoms is concerned, is effectual, is to fill the cavity with carbonate of soda, which affords relief, probably from its taking up any acid which may lie or be generated in the cavity, a common cause of irritation of this delicate organ, and from its forming a temporary means of protection to the denuded pulp.

There is, however, another and almost equally prevalent kind of tooth-ache, arising from inflammation of the investing membrane of the organ, the peridental membrane, and to this we propose to give the name of peridentitis. This is characterised by a more diffused, and more enduring, though less intense pain, and is frequently termed rheumatic tooth-ache. The sensation is, in this case, referred to the roots or origin of the nerves which supply the teeth, and is again reverberated to other and more distant parts. The symptoms in this case, differ from those in that just described, sufficiently to prevent any confusion, as to the source of the pain. There is more or less redness and tumefaction of the gums, around the affected tooth, to lead to its detection, although, as is not unfrequently the case, the pain be referred to the opposite row. There is also some tenderness, which may be discovered by making pressure, either directly upwards, or inwards and outwards. The paroxysm is not brought on by touching with the probe or otherwise, any particular spot in the cavity, and the pain, which is continuous, is not excited during mastication, nor on exposure to cold, but is, usually, exacerbated on coming into a warm room, or after fasting for some hours. If it have existed for some time, the tooth feels longer, and a sensation of numbness, or of positive tenderness, is experienced on closing the mouth. This is referable to inflammation, accompanied, if chronic, with thickening of the root, and must be treated by free depletion, one or two small leeches applied to the gum, or warm fomentations, with minute doses of blue pill and James' powder, determining an action to the skin will be found useful, after which, the tooth, if carious, as it will usually be found, must be filled up. A tooth, however, which is perfectly sound, or which has been well filled, may become subject to this affection, under an attack of indigestion or cold. The habitual use of a hard tooth-brush over the gums, in the direction of the root, is to be recommended, with the tincture of rhatony and camphor julep, with a little laudanum as a sedative. This affection is frequently the result of long neglected exposure of the pulp, although acute inflammation of the latter structure is, we believe, seldom if ever found in conjunction with inflammation of the peridental membrane. Where a repugnance is felt to the application of leeches, or where, from peculiar idiosyncrasy, erisypelatous symptoms forbid their use, (a circumstance which it is always desirable to ascertain,) the repeated application of some stimulating tincture to the gums, will, by a process of counter-irritation, withdraw inflammation from the more deep-seated structure.

Thus, then, with the present object of simplifying the subject of odontalgia, we confine our attention to two distinct kinds of tooth-ache, different alike in character and symptoms, different in the structures from which they arise, and requiring diverse and opposite kinds of treatment; the one arising from exposure or injury of the internal pulp, and calling for the exhibition of active remedies; the other proceeding from inflammation of the membrane of the root, and amenable to the lancet, leeching, fomentation, diaphoretics, and the ordinary measures for depletion. The pain in the former, confined to a minute spot in the carious cavity, and acceding in violent paroxysms of limited duration; in the latter being continuous, diffused, and having remote and anomalous sympathetic relations. The first being subdued, and temporarily removed by powerful stimulants, which would only have the effect of increasing the irritation and pain of the last. Let this grand broad distinction be kept in view, and let the symptoms in each individual case be made the subject of careful and considerate diagnosis, and the treatment of this prevalent and distressing malady will no longer be left in the hands of unprincipled empirics, nor be regarded, as it now justly is, the opprobrium medicorum.

[*London Forceps.*

